# Elucidation of the Effects of Si-Wu Tang on Menstrual Disorder Patterns through Activation of Aromatase and Antioxidation

**DOI:** 10.1155/2019/4761651

**Published:** 2019-03-05

**Authors:** Guan-Cheng Huang, Yi-Zhe Tsai, Chia-Jung Lee, Heng-Yu Chang, Ching-Chiung Wang

**Affiliations:** ^1^Division of Hemato-Oncology, Department of Internal Medicine, Yuan's General Hospital, Kaohsiung City 802, Taiwan; ^2^Program of Health-Business Administration, School of Nursing, Fooyin University, Kaohsiung City 831, Taiwan; ^3^Graduate Institute of Pharmacognosy, College of Pharmacy, Taipei Medical University, Taipei 110, Taiwan; ^4^PhD Program for Clinical Drug Discovery of Chinese Herbal Medicine, College of Pharmacy, Taipei Medical University, Taipei 110, Taiwan; ^5^Traditional Herbal Medicine Research Center, Taipei Medical University Hospital, Taipei 110, Taiwan; ^6^School of Medicine, College of Medicine, Taipei Medical University, Taipei 110, Taiwan; ^7^School of Pharmacy, College of Pharmacy, Taipei Medical University, Taipei 110, Taiwan

## Abstract

Si-Wu Tang (SWT), a traditional Chinese formula, is commonly used for treating female diseases, such as relief of menstrual discomfort and climacteric syndrome. The aim of this study was to explore the synergistic effects between each herb in SWT on menstrual disorder patterns. Estradiol regulation and antioxidative effects were indicators that ameliorated menstrual disorder patterns and the total polyphenol and polysaccharide contents were quality markers. According to relationships of bioactivity and phytochemical contents, we discuss the effects of each herb in SWT. In a testosterone-treated MCF-7 cell model,* Rehmannia glutinosa* and catalpol significantly increased the estradiol content and aromatase upregulation in cell culture. We suggest that catalpol is an aromatase promoter in SWT, and* R*.* glutinosa* is a major actor. In terms of the antioxidant activity, pentagalloylglucose, gallic acid, and ferulic acid had stronger antioxidative effects than other compounds. We suggest that the antioxidative ability depends on polyphenols, and* Paeonia lactiflora* is a major contributor. Based on the prescribing principle of traditional Chinese medicine (TCM) theory, we suggest that* R*.* glutinosa* in SWT act as an aromatase promoter in the role of sovereign for ameliorating menstrual disorder patterns. As* P*.* lactiflora* has the strongest antioxidant effects and can prevent ROS damage ovarian; therefore,* P*.* lactiflora* could help* R*.* glutinosa* work as a minister for menstrual disorder patterns and* R. glutinosa* and* P. lactiflora* are a herbal pair in SWT.

## 1. Introduction

Si-Wu-Tang (SWT), a traditional Chinese formula consisting of Rehmanniae Radix (the processed root of* Rehmannia glutinosa *Libosch,* Shu Di Huang*), Angelica Radix (the root of* Angelica sinensis *Diels,* Dang Gui*), Chuanxiong Rhizoma (the rhizome of* Ligusticum chuanxiong *Hortorum,* Chuan Xiong*), and Paeoniae Radix (the root of* Paeonia lactiflora *Pall,* Shao Yao*), was first recorded in* Tai Ping Huei Min Ho Chi Chu Fang* of the Sung dynasty (A.D. 1107~1110). It has traditionally been used for treating gynecological diseases, such as relief of menstrual irregularity, dysmenorrhea, uterine bleeding, climacteric syndrome, and other estrogen-related diseases. Furthermore, traditional medicine doctors also use it to cure weakness in various parts of the body due to anemia [1]. Recently, there have been many systemic biology-based investigations that demonstrated the mechanisms of traditional Chinese medicines (TCMs). The pharmacology of SWT may shed light on drug discovery for gynecological diseases as reported by a TCM integrative database analysis [2]. A Connectivity Map (CMAP) database analysis indicated that SWT has a phytoestrogenic effect as indicated by estradiol-treated MCF cells [3]. Moreover, microarray gene expression profiles of SWT in MCF cells were similar to the effects of estradiol, but it did not induce the oncogenes,* MYBL1* and* RET* [4]. Among its four constituent herbs, only Paeoniae Radix did not significantly increase the formation of a functional ER-ERE complex in response [4]. According to a population-based correlational study, the bodily pain of postpartum women who used SWT more than 10 times was more relieved than in those who did not use it [5]. On the other hand, SWT showed antioxidative effects through upregulating nuclear factor erythroid factor 2 (Nrf2) and z-ligustilide which is in Angelica Radix, and Chuanxiong Rhizoma could also activate Nrf2 [3, 6]. Moreover, a randomized, double-blind, placebo-controlled clinical trial offered the conclusion that oral administration of SWT for 6 months in healthy volunteers decreased serum oxidation and improved the lipid profile [7]. According to the above literature reviews, we targeted ovarian follicular maldevelopment to explore interactions of each herb in SWT to discuss the menstrual-regulating mechanism of SWT.

Si-Wu Tang (SWT), a traditional Chinese formula, is commonly used for treating female diseases, such as relief of menstrual discomfort and climacteric syndrome. According to the prescribing principle of TCM theory, a TCM formula becomes synergistic, more than the mere sum of its herbs. In this sense, the ingredients of a TCM formula have different roles, such as sovereign, minister, assistant, and courier. Therefore, we used subtracted one herb in SWT extracts to compare their bioactivity and explore the role of each component in SWT on menstrual disorder patterns. It was expected to clarify the prescribing principle of TCM theory of each herb in SWT based on the pharmacological effects.

## 2. Materials and Methods

### 2.1. Preparation of SWT and Its Components

Rehmanniae Radix (Shu Di Huang), Angelica Radix (Dang Gui), Chuanxiong Rhizoma (Chuan Xiong), and Paeoniae Radix (Shao Yao) were purchased from Sun Tan Pharmaceutical (New Taipei City, Taiwan). The medicinal materials were authenticated by the nonprofit organization, Brion Research Institute of Taiwan (New Taipei City, Taiwan).

The prescription of SWT was based on the unified formula announced by the Committee on Chinese Medicine and Pharmacy of the Department of Health (Taipei, Taiwan). The prescription of SWT includes four herbs in a ratio of 1:1:1:1 as shown in Table 1 [8]. Specifically SWT was immersed in a 20-fold amount of distilled water and boiled in a herb-extracting machine until half of the original amount of water was left. The extract was then filtered and freeze-dried. SWT without one herb (SWT without Dang Gui; SWT without Shao Yao; SWT without Chuan Xiong; SWT without Shu Di Huang) and each herb individually (Dang Gui, Shao Yao, Chuan Xiong and Shu Di Huang) were also prepared using the above-described extraction method. The yield of the nine extracts was about 25.2%~69.8% (w/w) (Table 2). The freeze-dried sample powder was stored at -20°C until use.

### 2.2. Phytochemical Analysis

#### 2.2.1. Total Polyphenol Analysis

The total phenol content was detected by the Folin-Ciocalteau method [9, 10]. SWT and SWT without individual herbal extracts were dissolved in double-distilled (dd) H_2_O. The sample solution was mixed with Folin-Ciocalteau reagent and a 7.5% aqueous Na_2_CO_3_ solution. After standing for 5 min at 50°C, the absorbance was measured at 600 nm against water on a *μ*Quant microplate reader. The amount of total phenols was expressed as gallic acid equivalents (mg GA/g sample) through a calibration curve prepared from standard amounts of gallic acid of 3.9~500.0 *μ*g/mL (Table 3).

#### 2.2.2. Total Polysaccharide Analysis

The total polysaccharide content was determined by a phenol-sulfuric method [11]. SWT and its component extracts were dissolved in ddH_2_O, and the sample solutions were mixed with 95% EtOH. After being allowed to stand for 30 min at room temperature, the precipitate was collected, and a 5% phenol solution and 2 M sulfuric acid were added. The well-mixed solution was shaken for 30 min, and its absorption was measured at 485 nm against water on a *μ*Quant microplate reader. The amount of total polysaccharide was expressed as glucose equivalents (mg glucose/g sample) using a calibration curve.

#### 2.2.3. Ferulic Acid and Gallic Acid Analysis

A high-performance liquid chromatographic (HPLC) system consisted of a Shimadzu (Kyoto, Japan) LC-10ATvp liquid chromatograph equipped with a DGU-14A degasser, an FCV-10ALvp low-pressure gradient flow control valve, an SIL-10ADvp autoinjector, an SPD-M10Avp diode array detector, and an SCL-10Avp system controller. Peak areas were calculated with Shimadzu Class-VP software (vers. 6.12 sp5). A TSK-gel® ODS-80TM column (5 *μ*m, 250 × 4 mm I.D.) (TOSHO®, Tokyo, Japan) was used. Gallic acid and ferulic acid were accurately weighed, dissolved, and double-diluted in HPLC-grade methanol to give serial concentrations in the range of 15.625~500 *μ*g/mL and 6.25~500 *μ*g/mL, respectively. The HPLC profiles of SWT and SWT without individual component herbs were analyzed with a mobile-phase system of 0.05% trifluoroacetic acid-acetonitrile (v/v) at 62: 38 and detected at 220 nm for gallic acid and 320 nm for ferulic acid. The analysis involved 10 *μ*L of sample solution. The operation was carried out at an oven temperature of 40°C. Calibration curves were plotted after a linear regression of the peak areas.

### 2.3. Analysis of Estradiol's Regulatory Effects

MCF-7 cells (human breast adenocarcinoma cell line) were obtained from the Food Industry Research and Development Institute (BCRC 60436l; Hsinchu, Taiwan) and were cultured in alpha-minimum essential medium containing 10% heat-inactivated fetal bovine serum (GIBCO, Grand Island, NY, USA) and 50 units/mL penicillin-streptomycin (GIBCO, Grand Island, NY, USA). The culture dishes were incubated at 37°C in a humidified incubator containing 5% CO_2_, and the medium was changed every 2 days.

MCF-7 cells were seeded in six-well plates, with each well containing 8×10^5^ cells, and cultured for 24 h. Cells in each dish were washed with phosphate-buffered saline (PBS), and new medium containing 10 *μ*g/mL testosterone and a test sample were added. After 24 h, the 17*β*-estradiol level of the supernatant was analyzed by an enzyme immunoassay (EIA) kit, and aromatase (CYP-19) expression of the cell pellet was analyzed by Western blotting. Proteins (30 *μ*g) were transferred to nitrocellulose membranes which were probed with antibodies specific for CYP-19 (SC-14244, Santa Cruz Biotechnology, Santa Cruz, CA, USA) and GAPDH (SC-32233, Santa Cruz) and then visualized with a BCIP/KBT kit (Sigma, St. Louis, MO, USA).

### 2.4. 1-Diphenyl-2-Picrylhydrazyl Radical (DPPH^.^)-Scavenging Analysis

Test samples were dissolved in ddH_2_O at 5 mg/mL and mixed with an equal volume of a 50 *μ*M DPPH^.^ solution in ethanol. The mixed solutions were kept in the dark at room temperature. After 30 min, the optical density of test samples was measured at 530 nm on a *μ*Quant microplate reader (BioTek, VT, USA). The DPPH^.^-scavenging rate (%) of test samples was calculated according to the following equation: [1 − (Ts/C)] × 100, where Ts and C are the optical density values of the test sample and control, respectively. Fifty percent inhibitory concentration (IC_50_) values, the concentration of each sample required to scavenge 50% of the DPPH^.^, were calculated from regression lines. Vitamin C was used as a positive control for the DPPH^.^-scavenging analysis.

### 2.5. Lipid Peroxidation Assay

Ovary tissues were obtained from Sprague-Dawley (SD) rats and homogenized in PBS. This homogenized solution was centrifuged for 10 min at 1200 rpm. The protein level in the homogenized tissue was quantified with Bioquant (Merck, Darmstadt, Germany). The homogenized tissue was treated with 125 mM tert-butyl hydroperoxide (TBH) with or without a test sample and eventually reacted with thiobarbituric acid (TBA) to form the pink adducts of malondialdehyde (MDA). The optical density of the sample solution was measured at 530 nm with a *μ*Quant spectrophotometer (BioTek). All rats used in this experiment were cared for according to ethical regulations on animal research of our university (permit no.: LAC-2017-0295).

### 2.6. Statistical Analysis

Results are presented as the mean ± standard deviation of three independent experiments. A one-way analysis of variance (ANOVA) in SPSS vers. 12 software (SPSS, Chicago, IL, USA) was used to analyze the results. Results were considered statistically significant at *p* < 0.05.

## 3. Results

### 3.1. Phytochemical Contents of SWT and Its Components

**Figure 1:**
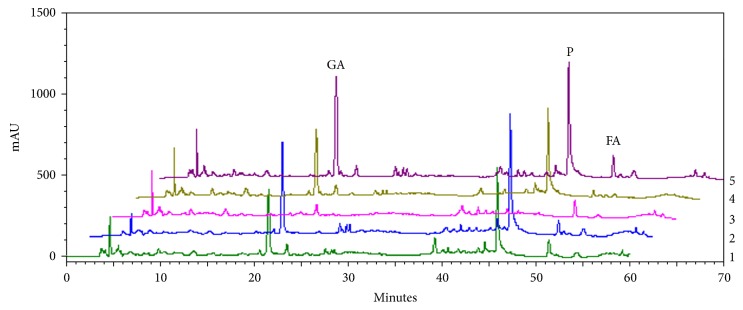
HPLC profiles of Si-Wu Tang (SWT) and its components. 1, SWT; 2, SWT without Dang Gui; 3, SWT without Shao Yao; 4, SWT without Chuan Xiong; 5, SWT without Shu Di Huang. Gallic acid (GA) retention time (Rt): 21.2 min; paeoniflorin (P) Rt: 46.4 min; ferulic acid (FA) Rt: 51.3 min.

The HPLC profiles of SWT and its components showed that gallic acid (retention time (Rt): 21.2 min) and paeoniflorin (Rt: 46.4 min) were major peaks followed by ferulic acid (Rt: 51.3 min). When SWT was lacking Shao Yao (3), gallic acid (Rt: 21.2 min) and paeoniflorin (Rt: 46.4 min) were not detected. Shao Yao is rich in gallic acid and paeoniflorin (Figure 1). Moreover, ferulic acid was the principal component of Dang Gui and Chuan Xiong. When SWT was lacking Chuan Xiong (4), the ferulic acid content was least in sample 4, followed by SWT lacking Dang Gui (2) (Table 3). The total polyphenol contents of SWT and its component extracts were analyzed by the Folin-Ciocalteau method. Sample 2 contained the most polyphenolic compounds and sample 3 the least (Table 3). According to the above results, we found the total phenolic contents of sample 5 to be greater than those of samples 2, 1, 4, and 3, sequentially (Table 3). Therefore, those results suggest that Shao Yao was a major contributor to the polyphenol content.

On the other hand, the total polysaccharide of samples was measured by the phenol-sulfuric method. Table 3 shows that sample 3 contained the most total polysaccharides and sample 5 the least. Sample 5 is STW without Shu Di Huang, and it can be inferred that the polysaccharide content of Shu Di Huang is highest in the four herbs. Therefore, having SWT without Dang Gui (2), without Shao Yao (3), or without Chuan Xiong (4), the proportion of Shu Di Huang in the prescription was increased, and the polysaccharide contents of samples 2, 3, and 4 were relatively increased. Therefore, we concluded that Shu Di Huang was a major contributor to the polysaccharide content.

### 3.2. Estradiol Upregulation Effects of SWT and Its Components in a Testosterone-Stimulated MCF-7 Cell Model

**Figure 2:**
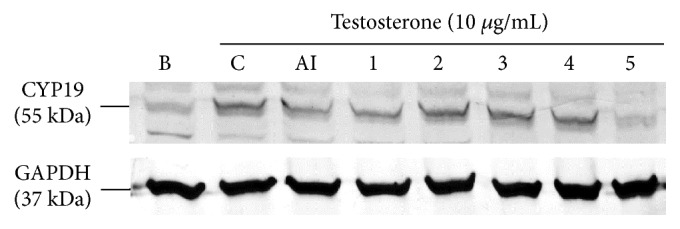
Regulation of aromatase (CYP-19) protein expression by Si-Wu Tang (SWT) and its components. B, blank, C, control; AI, anastrozole (100 *μ*M); 1, SWT; 2, SWT without Dang Gui; 3, SWT without Shao Yao; 4, SWT without Chuan Xiong; 5, SWT without Shu Di Huang. The concentration of all test samples was 200 *μ*g/mL.

**Figure 3:**
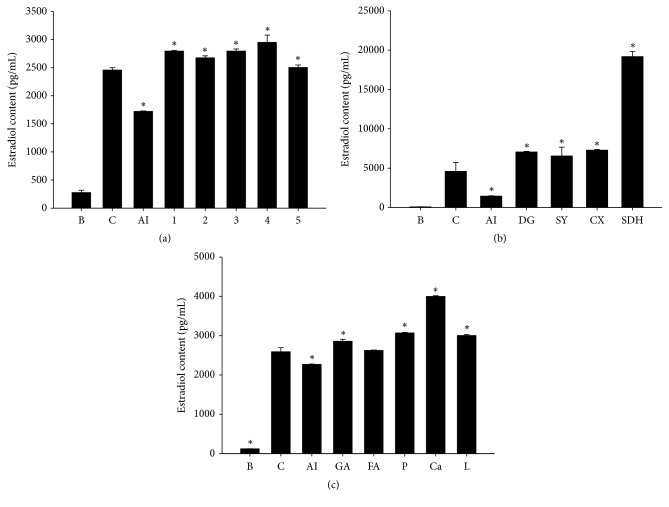
Stimulation of levels of estradiol in testosterone-treated MCF-7 cells after treatment with Si-Wu Tang (SWT) and its components. (a) SWT and SWT without one herb at 200 *μ*g/mL. 1, SWT; 2, SWT without Dang Gui; 3, SWT without Shao Yao; 4, SWT without Chuan Xiong; 5, SWT without Shu Di Huang. (b) The four herbs of SWT at 400 *μ*g/mL. DG, Dang Gui; SY, Shao Yao; CX, Chuan Xiong (3); SDH, Shu Di Huang. (c) Marker substances of SWT at 50 *μ*M. B, blank; C, testosterone only; AI, anastrozole; GA, gallic acid; FA, ferulic acid; P, paeoniflorin; Ca, catalpol; L, ligustrazine. B, blank, C, testosterone only (10 *μ*g/mL); AI, anastrozole (100 *μ*M), *∗* Compared to the control *p* < 0.05.

Aromatase is an enzyme that mediates biogenesis of estradiol levels in vivo, and MCF 7 cells contain aromatase. Testosterone was used as a substance, and it was added to MCF-7 cells and converted to estradiol which was used as an index marker [12]. First, we examined the expression of aromatase in MCF-7 cells with SWT or its components.  Figure 2 shows that sample 5 more significantly inhibited aromatase expression than anastrozole. Samples 2, 3, and 4 contained more Shu Di Huang contents than 1 and 5 and had greater aromatase expression. Second, the level of estradiol in the supernatant medium of MCF-7 cells was measured. Sample 5 and anastrozole significantly inhibited the estradiol content, while the other samples increased it (Figure 3(a)). Each individual component of SWT was added to MCF-7 cells, and all herbal extracts increased the estradiol content, with Shu Di Huang being the strongest (Figure 3(b)). Each one of the other herbs (Dang Gui, Shao Yao, and Chuan Xiong) exhibited no significant difference. Finally, the marker substances (gallic acid, ferulic acid, catalpol, and ligustrazine) of the four components were analyzed. Catapol, which is in Shu Di Huang, more significantly increased the estradiol content than did the others (Figure 3(c)). Among the marker substances, only ferulic acid, which is in Dang Gui and Chuan Xiong, did not significantly increase the estradiol content. The above results indicated that Shu Di Huang was the major component which could upregulate aromatase expression and the estradiol content, and the marker substance with upregulating effects was catalpol.

### 3.3. Antioxidant Effects of SWT and Its Components

**Figure 4:**
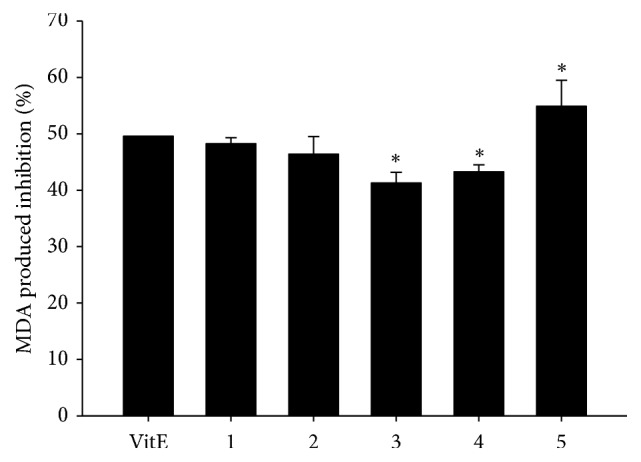
Inhibition of malondialdehyde (MDA) production by Si-Wu Tang (SWT) and its components in* t*-BuOOH-induced lipid peroxidation of rat ovary tissues. VitE, the concentration of vitamin E was 5 mM; the test concentrations of all samples were 200 *μ*g/mL. 1, SWT; 2, SWT without Dang Gui; 3, SWT without Shao Yao; 4, SWT without Chuan Xiong; 5, SWT without Shu Di Huang.

It was reported that oxidation could be one reason causing failure of ovary function [13]. Thus, this experiment was designed to examine whether SWT and its components mediate ovary function by altering the antioxidation activity. The DPPH radical-scavenging activities of SWT, its components, and marker substances were measured.  Table 4 shows that sample 3 had the least activity among the five samples; Shao Yao and 1,2,3,4,6-pentagalloylglucose had the strongest activities among the four herbs and marker substances, respectively. However, the other phenolic compounds, gallic acid and ferulic acid, also exhibited stronger scavenging activity than the others. Notably, when Shu Di Huang was absent from SWT, sample 5 could inhibit lipid oxidation in a TBH-induced ovary tissue model. However, samples 3 and 4 had no effects, when Shao Yao and Chuan Xiong were respectively absent from SWT (Figure 4). According to Figure 1 and Table 4, Shao Yao was the major antioxidant component, and the antioxidant marker substances were 1,2,3,4,6-pentagalloylglucose and gallic acid.

## 4. Discussion

A TCM prescription pattern is guided by the principle of treatment based on syndrome differentiation. Relationships among herbs comprising the formula include supplementing, opposing, and complementing each other. Individual herbs possess functions of principal, assistant, adjuvant, and dispatcher, respectively. The constituting herbs of a formula are called sovereign, minister, assistant, or courier [8]. According to the above TCM principles, the roles of the constituting herbs in SWT are as follows: Shu Di Huang is a sovereign, Dang Gui is a minister, while Shao Yao and Chuan Xiong are assistants (Table 1) [14]. Shu Di Huang is hematinic; Dang Gui regulates vitality and nourishes the blood; Shao Yao harmonizes the blood; Chuan Xiong activates blood flow and vitality. Therefore, SWT is applied to syndromes of blood deficiencies complicated by blood stasis [8]. However, it is not known whether the relative roles of the constituent herbs of SWT on menstrual disorder patterns are the same as for its blood-tonifying effects.

Ovarian follicular maldevelopment is an important pathogen-inducing menstrual disorder pattern. However, reactive oxygen species (ROS) and sex hormone disorders can induce ovarian follicular maldevelopment. Therefore, the antioxidative and estradiol-regulating effects of SWT, its constituent four herbs, and their marker substances would be indicator to define the relative roles of each herb in SWT. The results showed that Shu Di Huang acted as an aromatase promoter, and Shao Yao had the strongest antioxidant effects. Depending on amelioration of ovarian follicular maldevelopment through activation of aromatase and antioxidation, we recommend that Shu Di Huang could be a sovereign as a major worker, Shao Yao could be a minister to help Shu Di Huang, while Dang Gui and Chuan Xiong could be assistants. Shu Di Huang and Shao Yao, which both nourish the blood and yin, could be an herbal pair that protects the ovaries from menstrual disorder patterns. Ferulic acid is the marker substance for Dang Gui and Chuan Xiong which nourish and activate the blood, and the herbal pair acts like an energetic powerhouse to improve blood circulation [15]. In our previous study, we found that ferulic acid could enhance iron uptake and thus improve anemia [16]. Therefore, we suggest that the four herbs work together; Dang Gui and Chuan Xiong help Shu Di Huang and Shao Yao to improve menstrual disorder patterns through nourishing and activating the blood.

According to our results, Shu Di Huang was rich in polysaccharides and possessed activating effects on aromatase; thus, we suggest that polysaccharides are major contributors to these effects. In Figure 3, catalpol was an effective aromatase promoter. However, Shu Di Huang is deeply steam-processed Di Huang; catalpol levels gradually decreased [17]. The results indicated that Di Huang could be better than Shu Di Huang for activating aromatase's effects. On the other hand, Shao Yao was rich in polyphenol contents and antioxidants. Among the marker substances of Shao Yao, including gallic acid, 1,2,3,4,6-pentagalloylglucose, paeoniflorin, and paeonol, gallic acid and 1,2,3,4,6-pentagalloylglucose had greater antioxidative properties than paeoniflorin and paeonol (Table 4); gallic acid was richer than 1,2,3,4,6-pentagalloylglucose in Shao Yao (Figure 1). Therefore, we suggest that gallic acid is a quality maker substance of Shao Yao for its antioxidative effects. The other quality-marker substance was ferulic acid, which is an antioxidant and has high amounts in SWT, and could indicate Dang Gui and Chuan Xiong. Therefore, we used gallic acid and ferulic acid as quality-marker substances in SWT.

In summary, a TCM prescription pattern is like a concert. Each herb plays a beautiful song by itself, and together they make a symphony. The activities of Shu Di Huang, Shao Yao, Dang Gui, and Chuan Xiong exist by themselves, but when put together in SWT, the dosage ratio of each component is different and will cure different symptoms. In this study, we recommend that if the ratio of Shu Di Huang and Shao Yao should be increased, the function of SWT will be more forceful against menstrual disorder patterns through activating aromatase and antioxidative properties.

## Figures and Tables

**Table 1:** The formula of Si-Wu-Tang including its components and their functions.

Name (Chinese name)	Scientific Name (Family)	Substance markers	Dose (g)	Category of medicine	Role in the Formula
Rehmanniae (熟地黃, Shu Di Huang)	Root of *Rehmannia glutinosa *Libosch (Scrophulariaceae)	catalpol	7.5	Blood-tonifying medicinal	Sovereign

Angelicae (*當歸*, Dang Gui)	Root of *Angelica sinensis *Diels (Umbelliferae)	ferulic acid	7.5	Blood-tonifying medicinal	Minister

Paeoniae (*芍藥*, Shao Yao)	Root of *Paeonia lactiflora *Pall (Paeoniaceae)	gallic acid, paeoniflorin paeonol, 1,2,3,4,6-pentagalloylglucose	7.5	Blood-tonifying medicinal	Assistant

Chuanxiong (*川芎*, Chuan Xiong)	Root of *Ligusticum chuanxiong *Hortorum (Umbelliferae)	ferulic acid, ligustrazine	7.5	Blood-activating and stasis-resolving medicinal	Assistant

**Table 2:** The yield of the Si-Wu Tang (SWT) and its component extracts.

		SWT without one herb				Component herb of SWT			
Samples	1	2	3	4	5	Dang Gui	Shao Yao	Chuan Xiong	Shu Di Huang
Yield (%)	40.9	40.6	45.7	44.7	37.5	52.7	25.2	28.0	69.8

1, SWT; 2, SWT without Dang Gui; 3, SWT without Shao Yao; 4, SWT without Chuan Xiong; 5, SWT without Shu Di Huang.

**Table 3:** Phytochemical compositions of the Si-Wu Tang (SWT) and its component extracts.

Item	Total polyphenols	Total polysaccharides	Ferulic acid	Gallic acid
Concentration range (g/mL)	3.9~500.0^a^	0.0~500.0^b^	6.3~500.0	15.6~500.0

Linear equation	y=0.0041x+0.0006	y=0.7167x+0.076	y=130664x+1006583	y=61965x+415142

r^2^	0.9970	0.9997	0.9990	0.9990

*Content (mg/g)*				

1	9.9 ± 0.2	384.0 ± 8.4	0.5 ± 0.0	0.7 ± 0.0

2	11.2 ± 0.4	474.2 ± 7.3	0.4 ± 0.0	1.0 ± 0.0

3	9.3 ± 0.1	491.3 ± 10.9	0.5 ± 0.0	N.D.

4	9.7 ± 0.2	397.3 ± 5.4	0.2 ± 0.0	0.8 ± 0.0

5	10.5 ± 0.2	375.5 ± 11.0	0.7 ± 0.0	1.1 ± 0.0

ND, not detected; ^a^ the amount of total phenols is expressed as gallic acid equivalents; ^b^ the amount of total polysaccharide is expressed as glucose equivalents. 1, SWT; 2, SWT without Dang Gui; 3, SWT without Shao Yao; 4, SWT without Chuan Xiong; 5, SWT without Shu Di Huang.

**Table 4:** The DPPH radical-scavenging activity of Si-Wu Tang (SWT) and the marker substances of its components.

TCMs	Inhibition^a^ (%)	IC_50_ (mg/mL)
1	63.2 ± 1.3	0.7
2	70.4 ± 4.6	0.6
3	41.2 ± 0.9	1.4
4	62.5 ± 1.1	0.7
5	79.4 ± 0.6	0.4
Vitamin C (1.25 mM)	84.1 ± 0.8	0.04

Herbs	Inhibition^a^ (%)	IC_50_ (mg/mL)

Dang Gui	13.7 ± 0.3	-
Shao Yao	72.2 ± 2.1	0.2
Chuan Xiong	49.7 ± 1.4	1.0
Shu Di Huang	40.0 ± 2.0	-

Marker substances	Inhibition^a^ (%)	IC_50_ (*μ*M)

Ferulic acid	88.1 ± 0.5	267.0
Ligustrazine	0.4 ± 0.2	-
Gallic acid	87.5 ± 0.4	11.0
1,2,3,4,6-Pentagalloylglucose	89.2 ± 0.1	2.6
Paeoniflorin	14.3 ± 3.3	-
Paeonol	4.3 ± 0.8	-
Catapol	20.0 ± 1.7	-

^a^ The test concentration of samples was 1 mg/mL.

1, SWT; 2, SWT without Dang Gui; 3, SWT without Shao Yao; 4, SWT without Chuan Xiong; 5, SWT without Shu Di Huang; TCM, traditional Chinese medicine. -: value of IC_50_ was >1 mg/mL.

## Data Availability

The data used to support the findings of this study are included within the article.
